# 4-Nitro­benzyl 3,4-bis­(acet­yloxy)-2-(4-meth­oxy­phen­yl)pyrrolidine-1-carboxyl­ate: crystal structure, Hirshfeld surface analysis and computational chemistry

**DOI:** 10.1107/S2056989020007914

**Published:** 2020-06-16

**Authors:** Sofia Dallasta Pedroso, Ignez Caracelli, Julio Zukerman-Schpector, Monica Soto-Monsalve, Regina H. De Almeida Santos, Carlos Roque D. Correia, Ariel L. Llanes Garcia, Huey Chong Kwong, Edward R. T. Tiekink

**Affiliations:** a Laboratório de Cristalografia, Esterodinâmica e Modelagem Molecular, Departamento de Química, Universidade Federal de São Carlos, 13565-905 São Carlos, SP, Brazil; b Departmento de Física, Universidade Federal de São Carlos, 13565-905 São Carlos, SP, Brazil; c Instituto de Química de São Carlos, Universidade de São Paulo, São Carlos, SP, Brazil; d Instituto de Química, Universidade Estadual de Campinas, UNICAMP, C.P. 6154, CEP 13084-917 Campinas, Brazil; e Research Centre for Crystalline Materials, School of Science and Technology, Sunway University, 47500 Bandar Sunway, Selangor Darul Ehsan, Malaysia

**Keywords:** crystal structure, pyrrolidine, Hirshfeld surface analysis, NCI plots, computational chemistry

## Abstract

The title compound, C_23_H_24_N_2_O_9_, is a tetra-substituted pyrrolidine derivative with a twisted conformation, with the twist evident in the C—C bond bearing the adjacent acet­yloxy substituents. These are flanked on one side by a C-bound 4-meth­oxy­phen­yl group and on the other by a methyl­ene group. The almost *sp* ^2^-N atom [sum of angles = 357°] bears a 4-nitro­benzyl­oxycarbonyl substituent. In the crystal, ring-methyl­ene-C—H⋯O(acet­yloxy-carbon­yl) and methyl­ene-C—H⋯O(carbon­yl) inter­actions lead to supra­molecular layers lying parallel to (01); the layers stack without directional inter­actions between them. The analysis of the calculated Hirshfeld surfaces indicates the combined importance of H⋯H (42.3%), H⋯O/O⋯H (37.3%) and H⋯C/C⋯H (14.9%) surface contacts. Further, the inter­action energies, largely dominated by the dispersive term, point to the stabilizing influence of H⋯H and O⋯O contacts in the inter-layer region.

## Abstract 2

The title compound, containing a tetra-substituted pyrrolidine ring, has an N-bound (equatorial) 4-nitro­phen­yl)ethyl­carboxyl­ate group with an adjacent C-bound 4-meth­oxy­phenyl (bis­ectional) and then two acet­yloxy subtituents in equatorial and axial positions, respectively. The five-membered ring is twisted about the bond bearing the acet­yloxy subtituents.

## Chemical context   

The structure of the title tetra-substituted pyrrolidine deriv­ative, (I), was determined in connection with our on-going structural studies characterizing key synthetic inter­mediates in the synthesis of various α-glucosidase inhibitors (Zukerman-Schpector *et al.*, 2017 ▸; Dallasta Pedroso *et al.*, 2020 ▸). α-Glucosidase inhibitors are an important class of drugs employed in the treatment of a variety of diseases such as cancer, cystic fibrosis, diabetes and influenza (Kiappes *et al.*, 2018 ▸; Dhameja & Gupta, 2019 ▸).
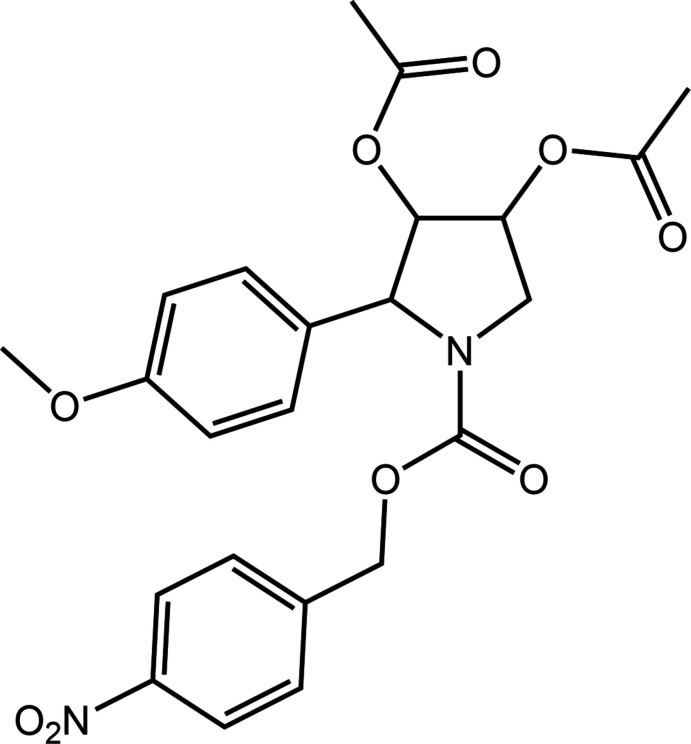


More specifically, (I) was generated during a study designed to synthesize the hy­droxy­lated proline derivative, (2*R*,3*S*,4*R*)-3,4-di­hydroxy­pyrrolidine-2-carb­oxy­lic acid, (II) (Garcia, 2008 ▸). In addition to being an α-glucosidase inhibitor, (II) is also found as a sub-structure of natural bioactive compounds such as, for example, a component of the repeated deca-peptide sequence of the adhesive protein *Mytilus edulis foot protein 1* (Mefp1), which is produced by the marine mussel *Mytilus edulis* and is responsible for the fixation capacity of the mussel to rock (Taylor & Weir, 2000 ▸). The synthetic study determined that in the final stages of the reaction sequence towards (II), it was not possible to smoothly remove the N-bound 4-nitro­benzyl­oxycarbonyl (PNZ) protecting group *via* catalytic hydrogenation as the ensuing mixture was difficult to purify. Therefore, it proved necessary to remove the PNZ protecting group through acid hydrolysis at reflux temperature, resulting in a low overall yield (34%) suggesting that there was no advantage in using PNZ.

The crystal and mol­ecular structures of (I) are described herein with this experimental study complemented by a detailed analysis of the mol­ecular packing by a combination of Hirshfeld surface analysis, non-covalent inter­action plots and computational chemistry.

## Structural commentary   

**Figure 1:**
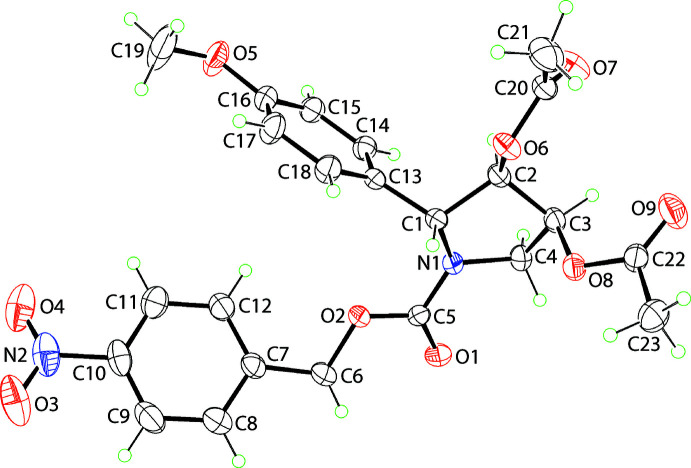
The mol­ecular structure of (I), showing the atom-labelling scheme and displacement ellipsoids at the 35% probability level.

The mol­ecular structure of (I), Fig. 1 ▸, is constructed about a tetra-substituted pyrrolidine ring with a N1-bound (4-nitro­phen­yl)ethyl­carboxyl­ate group and, respectively, C1–C3-bound 4-meth­oxy­phenyl, acet­yloxy and acet­yloxy substituents. For the illustrated mol­ecule, Fig. 1 ▸, the chirality of the C1–C3 atoms follows the sequence *R*, *R* and *S*, but it is noted that due crystal symmetry, the centrosymmetric unit cell contains equal numbers of the enanti­omers. The conformation of the five-membered ring is twisted about the C2—C3 bond with the C1—C2—C3—C4 torsion angle being 39.70 (16)°, consistent with a (+)*syn*-clinal configuration. The sum of the angles about the N1 atom is 356.7°, indicating an approximate *sp* ^2^ centre. The N1-bound group occupies an equatorial position with those at the C1–C3 centres being bis­ectional, equatorial and axial, respectively (Spek, 2020 ▸). When viewed towards the approximate plane through the pyrrolidine ring, the N-bound carboxyl­ate group is approximately co-planar, *i.e*. excluding the nitro­benzene residue. The C1-substituent lies to the opposite side of the plane than the C2 and C3-acet­yloxy groups; the dihedral angle between the acet­yloxy CO_2_ planes is 57.7 (2)°.

With respect to the least-squares plane through the pyrrolidine ring, the nitro­benzene and meth­oxy­benzene rings are splayed, as seen in the dihedral angles of 58.58 (8) and 77.65 (6)°, respectively; the dihedral angle between the benzene rings is 50.56 (5)°. There is a twist in the nitro­benzene ring as seen in the value of the C11—C10—N2—O4 torsion angle of 17.7 (3)°. By contrast, the meth­oxy group is co-planar with the ring to which it is connected, as shown by the C15—C16—O5—C19 torsion angle of 176.2 (2)°.

## Supra­molecular features   

**Figure 2:**
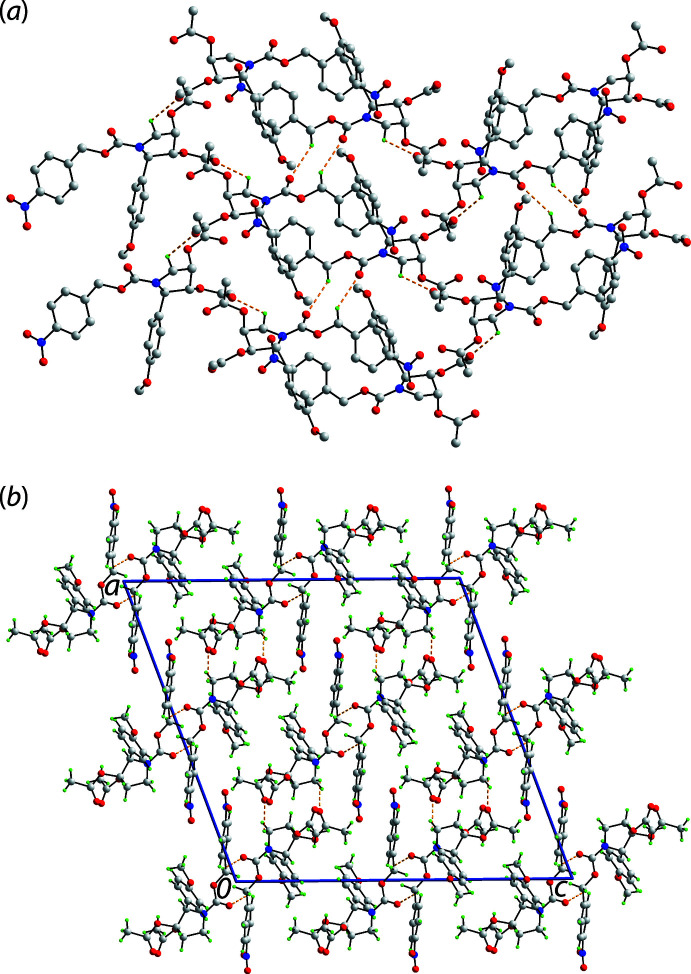
Mol­ecular packing in (I): (*a*) supra­molecular layer parallel to (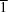
01) sustained by methyl­ene-C—H⋯O(carbon­yl) contacts shown as orange dashed lines (non-participating H atoms are omitted) and (*b*) view of the unit-cell contents shown in projection down the *b* axis.

The only directional non-covalent inter­actions of note in the crystal of (I) are two weak C—H⋯O contacts as listed in Table 1 ▸. The presence of ring-methyl­ene-C4—H⋯O7(acet­yloxy-carbon­yl) inter­actions lead to helical chains along the *b*-axis direction, being propagated by 2_1_ symmetry. The other inter­actions falling within the distance criteria of *PLATON* (Spek, 2020 ▸) are methyl­ene-C6—H⋯O1(carbon­yl) inter­actions, formed between centrosymmetrically related (4-nitro­phen­yl)ethyl­carboxyl­ate groups, which lead to the formation of ten-membered {⋯OCOCH}_2_ synthons. These serve to connect the helical chains into a layer lying parallel to (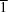
01), Fig. 2 ▸(*a*). A view of the unit-cell contents is shown in Fig. 2 ▸(*b*), highlighting the stacking of layers, without directional inter­actions between them.

## Non-covalent inter­action plots   

**Figure 3:**
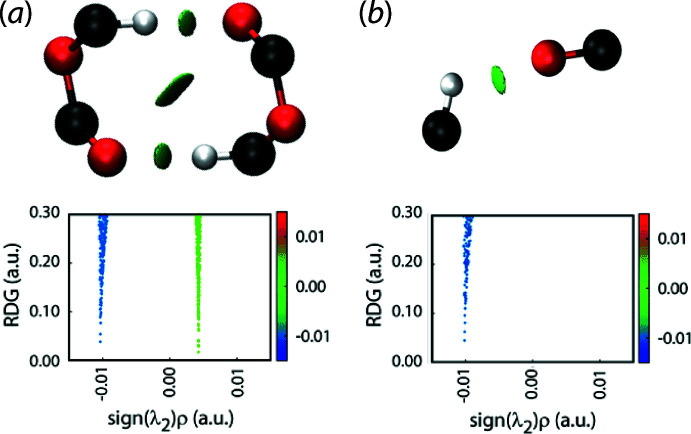
Non-covalent inter­action plots for the following inter­actions in (I): (*a*) methyl­ene-C6—H⋯O1(carbon­yl) and (*b*) ring-methyl­ene-C4—H⋯O7(acet­yloxy-carbon­yl).

The aforementioned weak C—H⋯O contacts identified in *Supra­molecular features* were also evaluated by calculating non-covalent inter­action plots (Johnson *et al.*, 2010 ▸; Contreras-García *et al.*, 2011 ▸). In short, these calculations indicate whether non-bonding contacts are attractive, weakly attractive or repulsive. The methyl­ene-C6—H⋯O1(carbon­yl) inter­actions giving rise to the ten-membered {⋯OCOCH}_2_ synthons are highlighted in the upper view of Fig. 3 ▸(*a*) with the green isosurface between the inter­acting atoms and the distinctive blue feature in the reduced density gradient (RDG) versus sign(λ^2^)*ρ*(*r*) plot in the lower view, *i.e*. indicating the density value is less than 0.0 a.u., suggest these inter­actions are weakly attractive. The same is true for the ring-methyl­ene-C4—H⋯O7(acet­yloxy-carbon­yl) inter­actions that lead to the helical chain, Fig. 3 ▸(*b*).

## Hirshfeld surface analysis   

**Figure 4:**
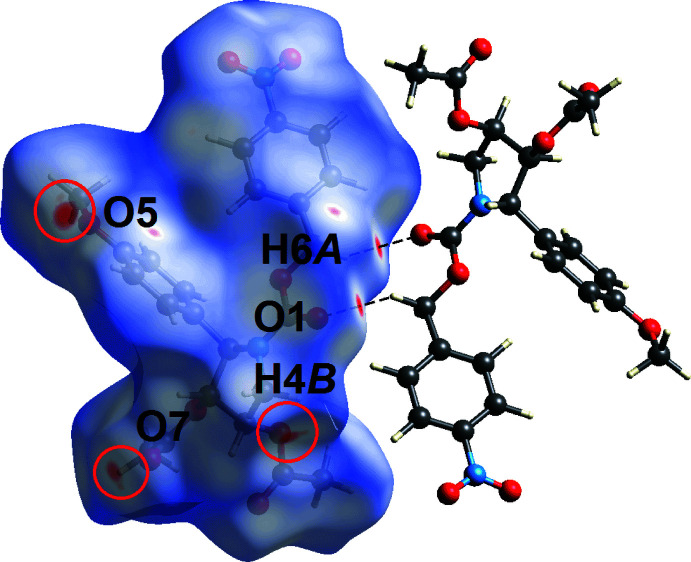
A view of the Hirshfeld surface mapped for (I) over *d*_norm_ in the range −0.090 to +1.583 arbitrary units showing the C—H⋯O inter­actions as black dashed lines.

The Hirshfeld surface analysis of (I) involved the calculation of the *d*_norm_-surface plots, electrostatic potential (calculated using the STO-3G basis set at the Hartree–Fock level of theory) and two-dimensional fingerprint plots following literature procedures (Tan *et al.*, 2019 ▸) using *Crystal Explorer 17* (Turner *et al.*, 2017 ▸). The weak methyl­ene-C6—H⋯O1(carbon­yl) inter­actions are reflected as bright-red spots near the methyl­ene-H6*A* and carbonyl-O1 atoms on the *d*_norm_-surface plot of (I) shown in Fig. 4 ▸. Additional diffuse red spots are also noted near the meth­oxy-O5 and carbonyl-O7 atoms in Fig. 4 ▸, which reflect their participation in short C5⋯O5 and C4⋯O7 contacts with separations ∼0.1 Å shorter than the sum of their van der Waals radii, Table 2 ▸. Further, faint spots near atom H4*B* as well as the O5 and O7 atoms (each difficult to discern in Fig. 4 ▸) are attributed to methyl­ene-C4—H4*B*⋯O7(carbon­yl) and O2⋯O5 short contacts, being ∼0.02 Å shorter than their respective sums of the van der Waals radii, Table 2 ▸.

**Figure 5:**
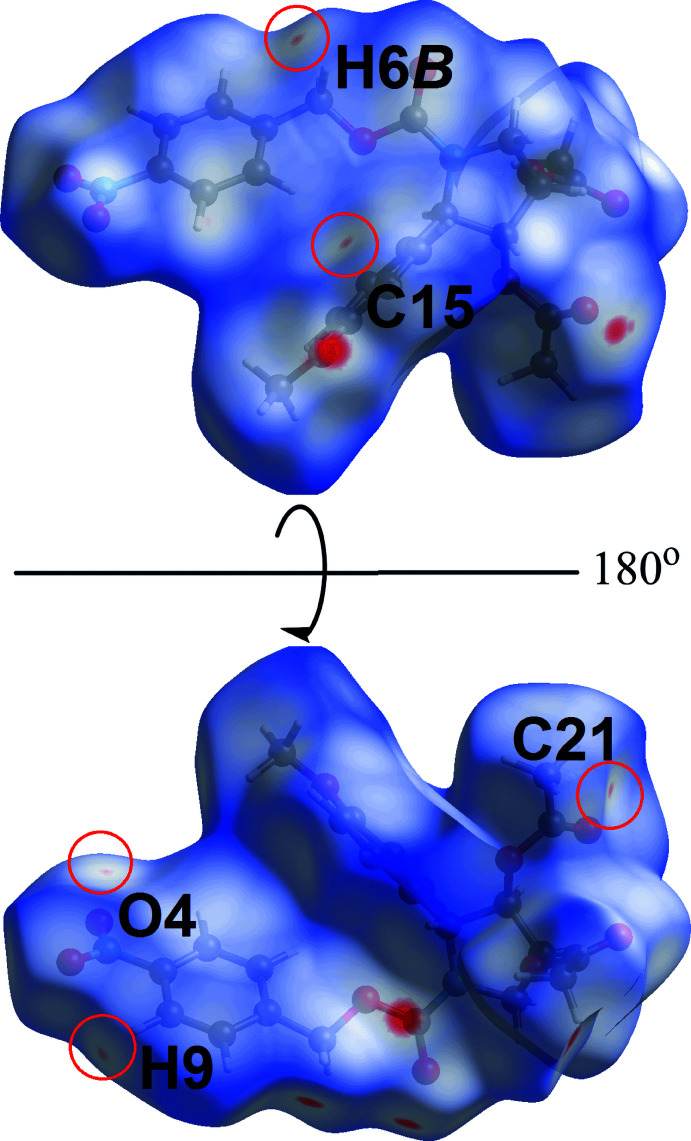
Two views of the Hirshfeld surface mapped over *d*_norm_ for (I) in the range −0.090 to +1.583 arbitrary units, highlighting evidence for long-range C—H⋯C inter­actions and O⋯O short contacts within red circles (see text).

**Figure 6:**
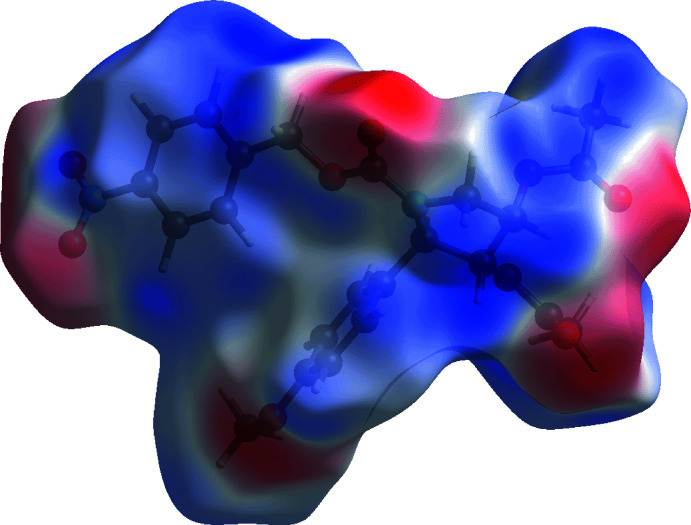
A view of the Hirshfeld surface mapped over the calculated electrostatic potential for (I). The potentials were calculated using the STO-3 G basis set at the Hartree–Fock level of theory over a range of −0.067 to 0.040 a.u. The red and blue regions represent negative and positive electrostatic potentials, respectively.

In the views of Fig. 5 ▸, the faint red spots that appear near the methyl­ene (H6*B*), benzyl (C15 and H9), methyl (C21) and nitro (O4) atoms correspond to long-range intra-layer methyl­ene-C6—H6*B*⋯C15(benz­yl), benzyl-C9—H9⋯C21(meth­yl) inter­actions and inter-layer O4⋯O4 short contacts, Table 2 ▸. The Hirshfeld surface mapped over the electrostatic potential in Fig. 6 ▸ highlights the donors and acceptors of the indicated inter­actions through blue (positive electrostatic potential) and red (negative electrostatic potential), respectively.

**Figure 7:**
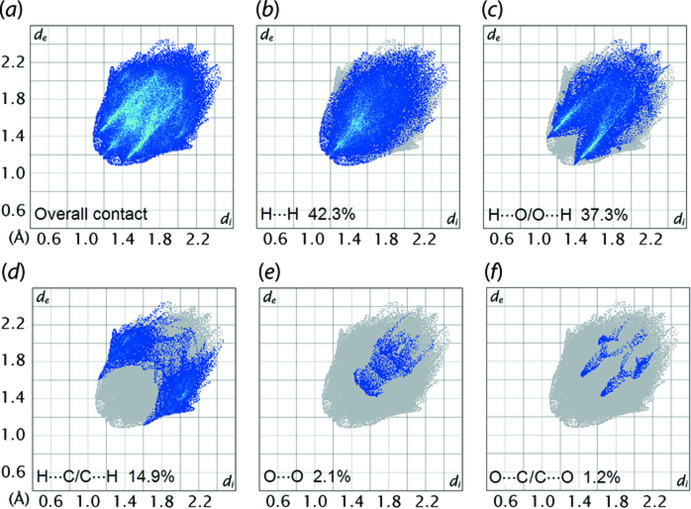
(*a*) The full two-dimensional fingerprint plot for (I) and (*b*)–(*f*) those delineated into H⋯H, H⋯O/O⋯H, H⋯C/C⋯H, O⋯O and O⋯C/C⋯O contacts, respectively.

As illustrated in Fig. 7 ▸(*a*), the two-dimensional fingerprint plot for the Hirshfeld surface of (I) is shown in the upper left and lower right sides of the *d*_e_ and *d*_i_ diagonal axes, and those delineated into H⋯H, H⋯O/O⋯H, H⋯C/C⋯H, O⋯O and O⋯C/C⋯O contacts are illustrated in Fig. 7 ▸(*b*)–(*f*), respectively. The percentage contributions from different inter­atomic contacts are summarized in Table 3 ▸. The H⋯H contacts contribute 42.3% to the overall Hirshfeld surface with the shortest contact, manifested in the round-shape peak tipped at *d*_e_ = *d*_i_ ∼2.4 Å, Fig. 7 ▸(*b*), corresponding to the H17⋯H23*B* inter-layer contact listed in Table 2 ▸. The H⋯O/O⋯H contacts contribute 37.3% to the overall Hirshfeld surface, reflecting the significant C—H⋯O contacts evident in the packing, Tables 1 ▸ and 2 ▸. The shortest contacts are reflected as two sharp spikes at *d*_e_ + *d*_i_ ∼2.5 Å in Fig. 7 ▸(*c*). The H⋯C/C⋯H contacts that match the long-range C—H⋯C inter­actions discussed above are shown as a pairs of forceps-like tips at *d*_e_ + *d*_i_ ∼2.7 Å in the fingerprint plot delineated into H⋯C/C⋯H contacts, Fig. 7 ▸(*d*). Although both O⋯O and O⋯C/C⋯O contacts appear at *d*_e_ + *d*_i_ ∼3.0 Å in the respective fingerprint plots, Fig. 7 ▸(*e*) and (*f*), their contributions to the overall Hirshfeld surface are only 2.1 and 1.2%, respectively. The other inter­atomic contacts have a negligible effect on the mol­ecular packing as their accumulated contribution is about 2.2%.

## Energy frameworks   

The pairwise inter­action energies between the mol­ecules in the crystal of (I) were calculated by summing up four energy components, comprising the electrostatic (*E*_ele_), polarization (*E*_pol_), dispersion (*E*_dis_) and exchange-repulsion (*E*_rep_) energies as per the literature (Turner *et al.*, 2017 ▸). In the present study, the energy framework of (I) was generated by employing the 6-31G(*d*,*p*) basis set with the B3LYP function. The individual energy components as well as the total inter­action energies are collated in Table 4 ▸. As anti­cipated, the dispersive component makes the major contribution to the inter­action energies owing to the absence of conventional hydrogen bonding in the crystal. The most significant stabilization energies are found in the intra-layer region and arise from the directional contacts outlined in *Hirshfeld surface analysis* as well as two additional C—H⋯O inter­actions, *i.e*. methyl­ene-C4—H4*A*⋯O4(nitro) and methyl-C21—H21*C*⋯O4(nitro) with H⋯O separations of 2.63 and 2.77 Å, respectively.

**Figure 8:**
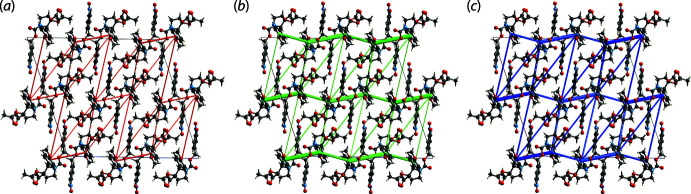
Perspective views of the energy frameworks calculated for (I) and viewed down the *b* axis showing (*a*) electrostatic potential force, (*b*) dispersion force and (*c*) total energy. The radii of the cylinders are proportional to the relative magnitudes of the corresponding energies and were adjusted to the same scale factor of 50 with a cut-off value of 5 kJ mol^−1^ within 1 × 1 × 1 unit cells.

The stabilization energies in the inter-layer region are also dominated by the *E*_dis_ terms associated with the H⋯H contacts as well as the long-range C—H⋯O inter­actions (−14.4 kJ mol^−1^). For the former, the maximum energy is not found for the shortest H17⋯H23*B* contact (−7.1 kJ mol^−1^), Table 2 ▸ and Fig. 8 ▸(*b*), but rather for a pair of benzene-H⋯H(meth­yl) inter­actions occurring in close proximity in a hydrogen-rich region but at longer separations (−34.2 kJ mol^−1^). For the inter-layer O4⋯O4 contact mentioned above, there are almost equal contributions from *E*_ele_ and *E*_dis_, Table 4 ▸, giving rise to a total inter­action energy of −27.7 kJ mol^−1^. The magnitudes of inter­molecular energies are represented graphically in Fig. 8 ▸, and clearly demonstrate the dominance of the *E*_dis_ in the mol­ecular packing.

## Database survey   

**Figure 9:**
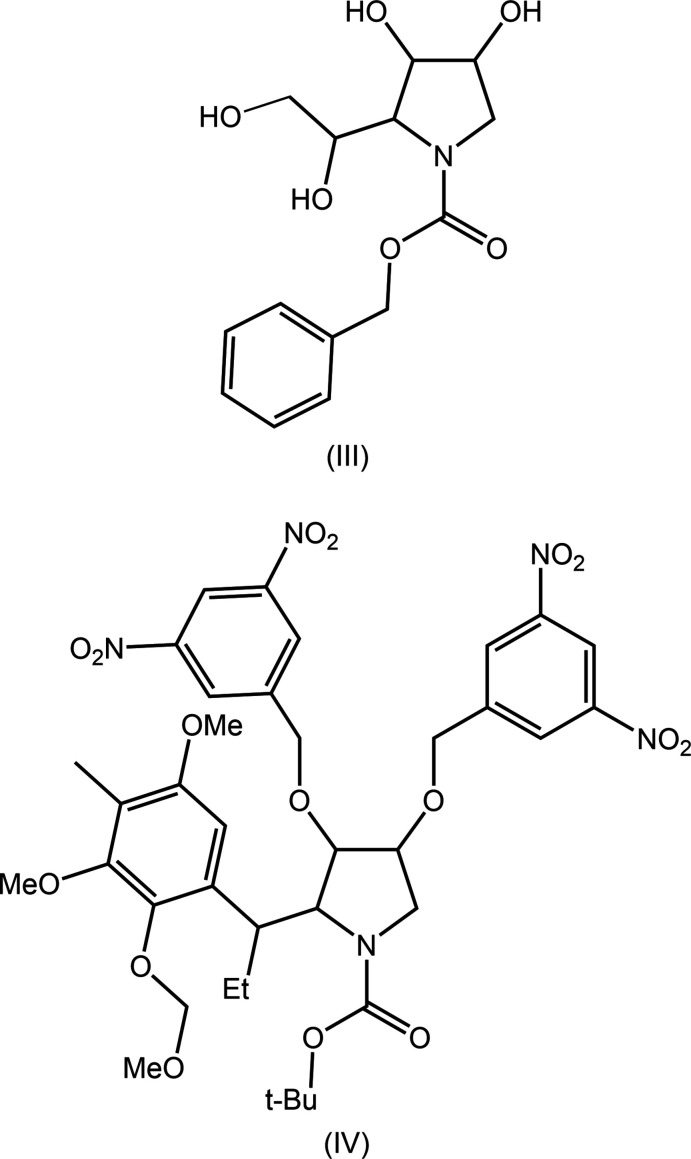
Chemical diagrams for (III) and (IV).

There are relatively few related structures having a similar substitution pattern to the tetra-substituted pyrrolidine ring of (I). The chemical diagrams for the two most closely related structures, (III), which has two hydroxyl substituents rather than acet­yloxy (ALAVOA; Qian *et al.*, 2016 ▸), and (IV), which has more complex substituents (RAJDUC; Coleman *et al.*, 2004 ▸), are shown in Fig. 9 ▸.

## Synthesis and crystallization   

To a solution of 4-nitro­benzyl (2*S*,3*S*,4*R*)-3,4-dihy­droxy-2-(4-meth­oxy­phen­yl)pyrrolidine-1-carboxyl­ate (602 mg, 1.55 mmol) in CH_2_Cl_2_ (15 ml) were added pyridine (0.80 ml, 18.584 mmol), acetic anhydride (3.00 ml, 31.8 mmol) and *N*,*N*-dimethyl-4-amino­pyridine (2.00 mg, 0.0164 mmol). The solution was stirred for 2 h at room temperature, concentrated in a rota-evaporator and the residue dissolved in EtOAc (10 ml). The resulting solution was washed with a HCl 5% solution (3 × 5 ml) and with saturated solutions of NaHCO_3_ (2 × 5 ml) and of NaCl (5 ml). The phases were separated and the organic phase was dried with anhydrous Na_2_SO_4_, filtered and concentrated *in vacuo*.

The residue was purified by flash column chromatography in silica gel, using an EtOAc/*n*-hexane elution gradient (1:3 and 1:2). Yield: 716 mg (98%). Colourless irregular crystals for the X-ray analysis were obtained by the slow evaporation of its *n*-hexane solution. M.p. 409.5–410.5 K. The ^1^H and ^13^C{^1^H} NMR reflect the presence of two conformational rotamers in solution. ^1^H NMR (500 MHz, C_6_D_6_): δ = 7.75 (*d*, *J* = 7.3 Hz, 0.4H); 7.65 (*d*, *J* = 7.9 Hz, 1.2H); 7.18 (*m*, 1.9H); 6.99 (*d*, *J* = 7.9 Hz, 1.1H); 6.76 (*d*, *J* = 7.0 Hz, 0.5H); 6.72 (*d*, *J* = 7.3 Hz, 0.6H); 6.65 (*d*, *J* = 7.9 Hz, 1.3H); 6.37 (*d*, *J* = 9.3 Hz, 1H); 5.42 (*s*, 0.2H); 5.33 (*m*, 1.9H); 5.00 (*s*, 0.5H); 4.92 (*d*, *J* = 13.7 Hz, 0.6H); 4.74 (*s*, 0.6H); 4.44 (*d*, *J* = 13.7 Hz, 0.6H); 3.89 (*m*, 1.8H); 3.72 (*s*, 0.3H); 3.29 (*s*, 3H); 3.35–3.23 (*m*, 0.3H); 1.61–1.60 (2*s*, 6H). ^1^H NMR (500 MHz, CDCl_3_, TMS r.t.): δ = 8.23 (*d*, *J* = 8.2 Hz, 0.6H); 8.00 (*d*, *J* = 8.2 Hz, 1.2H); 7.53 (*d*, *J* = 7.9 Hz, 0.7H); 7.16 (*m*, 2H); 6.96 (*d*, *J* = 8.5 Hz, 1.2H); 6.88 (*d*, *J* = 8.5 Hz, 2.0H); 5.45–5.32 (*m*, 1H); 5.31–5.18 (*m*, 2.3H); 5.01–4.87 (*m*, 1.6H); 4.13 (*m*, 0.3H); 4.06 (*dd*, *J* = 11.6 Hz and 6.4 Hz, 0.7H); 3.85–3.67 (*s* + *m*, 4.1H); 2.12-2.07 (4*s*, 6H). ^13^C{^1^H} NMR (125 MHz, CDCl_3_, r.t.): δ = 169.9; 169.8; 159.4; 159.2; 154.2; 154.1; 147.6; 147.2; 143.6; 143.4; 130.6; 129.4; 128.1; 127.5; 126.8; 126.7; 123.7; 123.4; 114.2; 78.2; 69.2; 68.7; 65.7; 65.5; 64.7; 64.1; 55.3; 55.2; 49.0; 48.4; 20.8; 20.7; 20.6.

## Refinement details   

Crystal data, data collection and structure refinement details are summarized in Table 5 ▸. The carbon-bound H atoms were placed in calculated positions (C—H = 0.93–0.98 Å) and were included in the refinement in the riding model approximation, with *U*_iso_(H) set to 1.2–1.5*U*_eq_(C).

## Supplementary Material

Crystal structure: contains datablock(s) I, global. DOI: 10.1107/S2056989020007914/hb7923sup1.cif

Structure factors: contains datablock(s) I. DOI: 10.1107/S2056989020007914/hb7923Isup2.hkl

Supporting information file. DOI: 10.1107/S2056989020007914/hb7923Isup3.cml

CCDC reference: 2009242

Additional supporting information:  crystallographic information; 3D view; checkCIF report

## Figures and Tables

**Table 1:** Hydrogen-bond geometry (Å, °)

*D*—H⋯*A*	*D*—H	H⋯*A*	*D*⋯*A*	*D*—H⋯*A*
C4—H4*B*⋯O7^i^	0.97	2.60	3.129 (2)	115
C6—H6*A*⋯O1^ii^	0.97	2.54	3.250 (2)	130

Symmetry codes: (i) 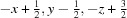; (ii) 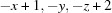.

**Table 2:** Summary of short inter­atomic contacts (Å) in (I)^*a*^

Contact	Distance	Symmetry operation
C6—H6*A*⋯O1^*b*^	2.47	−*x* + 1, −*y*, −*z* + 2
C4—H4*B*⋯O7^*b*^	2.55	−*x* + 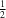 , *y* − 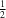 , −*z* + 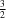
C4⋯O7	3.13	−*x* + 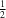 , *y* + 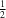 , −*z* + 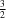
C5⋯O5	3.08	*x*, *y* − 1, *z*
O2⋯O5	3.02	*x*, *y* − 1, *z*
C6—H6*B*⋯C15	2.73	−*x* + 1, −*y* + 1, −*z* + 2
C9—H9⋯C21	2.75	*x* + 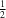 , −*y* + 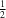 , *z* + 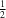
O4⋯O4	2.75	−*x* + 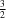 , −*y* + 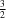 , −*z* + 2
H17⋯H23*B*	2.35	−*x* + 1, *y* + 1, −*z* + 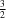

Notes: (*a*) The inter­atomic distances are calculated in *Crystal Explorer 17* (Turner *et al.*, 2017 ▸) whereby the *X*—H bond lengths are adjusted to their neutron values. (*b*) These inter­actions correspond to the inter­actions listed in Table 1 ▸.

**Table 3:** Percentage contributions of inter­atomic contacts to the Hirshfeld surface for (I)

Contact	Percentage contribution
H⋯H	42.3
H⋯O/O⋯H	37.3
H⋯C/C⋯H	14.9
O⋯O	2.1
O⋯C/C⋯O	1.2
Others	2.2

**Table 4:** Summary of inter­action energies (kJ mol^−1^) calculated for (I)

Contact	*R* (Å)	*E*_ele_	*E*_pol_	*E*_dis_	*E*_rep_	*E*_tot_
Intra-layer region						
C4—H4*B*⋯O7^i^ +						
C4⋯O7^i^	10.99	−17.8	−6.1	−29.1	18.3	−37.3
C6—H6*A*⋯O1^ii^	9.21	−23.8	−6.9	−23.2	21.7	−37.0
C5⋯O5^iii^ +						
O2⋯O5^iii^	8.29	−8.4	−2.7	−56.3	29.1	−41.8
C9—H9⋯C21^iv^	14.12	−12.7	−3.4	−20.5	12.0	−26.4
C6—H6*B*⋯C15^v^ +						
C4—H4*A*⋯O4^v^	6.55	−18.1	−4.5	−87.1	52.8	−65.8
C21—H21*C*⋯O4^vi^	15.04	−2.1	−1.0	−3.7	1.5	−5.2
Inter-layer region						
H17⋯H23*B* ^vii^	10.38	2.9	−1.2	−16.5	8.2	−7.1
H17⋯H21*B* ^vii^ +						
H18⋯H21*B* ^viii^	6.24	−1.1	−1.6	−52.9	23.0	−34.2
O4⋯O4^ix^	13.71	−16.1	−4.4	−16.2	10.8	−27.7
C8—H8⋯O3^*x*^	12.70	−5.4	−1.3	−10.2	1.9	−14.4

Symmetry codes: (i) −*x* + 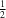, *y* − 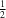, −*z* + 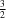; (ii) −*x* + 1, −*y*, −*z* + 2; (iii) *x*, *y* − 1, *z*; (iv) *x* + 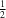, −*y* + 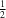, *z* + 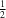; (v) −*x* + 1, −*y* + 1, −*z* + 2; (vi) *x* − 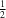, −*y* + 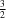, *z* − 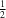; (vii) −*x* + 1, *y* + 1, − *z* + 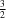; (viii) −*x* + 1, *y*, −*z* + 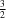; (ix) −*x* + 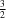, −*y* + 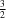, −*z* + 2; (*x*) −*x* + 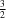, −*y* + 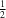, −*z* + 2.

**Table 5:** Experimental details

Crystal data	
Chemical formula	C_23_H_24_N_2_O_9_
*M*_r_	472.44
Crystal system, space group	Monoclinic, *C*2/*c*
Temperature (K)	293
*a*, *b*, *c* (Å)	23.6396 (5), 8.2906 (2), 24.7683 (5)
β (°)	110.013 (1)
*V* (Å^3^)	4561.13 (18)
*Z*	8
Radiation type	Mo *K*α
μ (mm^−1^)	0.11
Crystal size (mm)	0.40 × 0.36 × 0.18

Data collection	
Diffractometer	Enraf–Nonius TurboCAD-4
Absorption correction	Multi-scan (*SADABS*; Sheldrick, 1996 ▸)
*T*_min_, *T*_max_	0.686, 0.745
No. of measured, independent and observed [*I* > 2σ(*I*)] reflections	22357, 4172, 3646
*R*_int_	0.020
(sin θ/λ)_max_ (Å^−1^)	0.603

Refinement	
*R*[*F* ^2^ > 2σ(*F* ^2^)], *wR*(*F* ^2^), *S*	0.041, 0.112, 1.01
No. of reflections	4172
No. of parameters	310
H-atom treatment	H-atom parameters constrained
Δρ_max_, Δρ_min_ (e Å^−3^)	0.30, −0.22

Computer programs: *CAD-4 EXPRESS* (Enraf–Nonius, 1989 ▸), *XCAD4* (Harms & Wocadlo, 1995 ▸), *SIR2014* (Burla *et al.*, 2015 ▸), *SHELXL2018/3* (Sheldrick, 2015 ▸), *ORTEP-3 for Windows* (Farrugia, 2012 ▸), *MarvinSketch* (ChemAxon, 2010 ▸), *DIAMOND* (Brandenburg, 2006 ▸) and *publCIF* (Westrip, 2010 ▸).

## supplementary crystallographic information

### Crystal data

**Table:**

C_23_H_24_N_2_O_9_	*F*(000) = 1984
*M**_r_* = 472.44	*D*_x_ = 1.376 Mg m^−^^3^
Monoclinic, *C*2/*c*	Mo *K*α radiation, λ = 0.71073 Å
*a* = 23.6396 (5) Å	Cell parameters from 9984 reflections
*b* = 8.2906 (2) Å	θ = 2.6–25.4°
*c* = 24.7683 (5) Å	µ = 0.11 mm^−^^1^
β = 110.013 (1)°	*T* = 293 K
*V* = 4561.13 (18) Å^3^	Irregular, colourless
*Z* = 8	0.40 × 0.36 × 0.18 mm

### Data collection

**Table:**

Enraf–Nonius TurboCAD-4 diffractometer	*R*_int_ = 0.020
Radiation source: Enraf–Nonius FR590	θ_max_ = 25.4°, θ_min_ = 1.8°
non–profiled ω/2θ scans	*h* = −28→28
Absorption correction: multi-scan (SADABS; Sheldrick, 1996)	*k* = −7→10
*T*_min_ = 0.686, *T*_max_ = 0.745	*l* = −29→29
22357 measured reflections	3 standard reflections every 120 min
4172 independent reflections	intensity decay: 2%
3646 reflections with *I* > 2σ(*I*)	

### Refinement

**Table:**

Refinement on *F*^2^	Primary atom site location: structure-invariant direct methods
Least-squares matrix: full	Secondary atom site location: difference Fourier map
*R*[*F*^2^ > 2σ(*F*^2^)] = 0.041	Hydrogen site location: inferred from neighbouring sites
*wR*(*F*^2^) = 0.112	H-atom parameters constrained
*S* = 1.01	*w* = 1/[σ^2^(*F*_o_^2^) + (0.0475*P*)^2^ + 5.1476*P*] where *P* = (*F*_o_^2^ + 2*F*_c_^2^)/3
4172 reflections	(Δ/σ)_max_ = 0.001
310 parameters	Δρ_max_ = 0.30 e Å^−^^3^
0 restraints	Δρ_min_ = −0.22 e Å^−^^3^

### Special details

**Table:**

Geometry. All esds (except the esd in the dihedral angle between two l.s. planes) are estimated using the full covariance matrix. The cell esds are taken into account individually in the estimation of esds in distances, angles and torsion angles; correlations between esds in cell parameters are only used when they are defined by crystal symmetry. An approximate (isotropic) treatment of cell esds is used for estimating esds involving l.s. planes.

### Fractional atomic coordinates and isotropic or equivalent isotropic displacement parameters (Å^2^)

**Table:**

	*x*	*y*	*z*	*U*_iso_*/*U*_eq_	
C1	0.41109 (7)	0.33440 (18)	0.82213 (6)	0.0313 (3)	
H1	0.439778	0.265045	0.812398	0.038*	
C2	0.35064 (7)	0.33405 (19)	0.77230 (7)	0.0336 (4)	
H2	0.327740	0.430457	0.774943	0.040*	
C3	0.31777 (7)	0.1856 (2)	0.78170 (7)	0.0378 (4)	
H3	0.274273	0.192051	0.761254	0.045*	
C4	0.33330 (7)	0.1864 (2)	0.84615 (7)	0.0403 (4)	
H4A	0.304809	0.251133	0.857137	0.048*	
H4B	0.333567	0.077780	0.860767	0.048*	
C5	0.43296 (7)	0.20974 (18)	0.91863 (7)	0.0345 (4)	
C6	0.53264 (8)	0.2143 (2)	0.98048 (8)	0.0528 (5)	
H6A	0.542252	0.103154	0.974917	0.063*	
H6B	0.516931	0.217441	1.011878	0.063*	
C7	0.58827 (8)	0.3154 (2)	0.99508 (7)	0.0417 (4)	
C8	0.64314 (9)	0.2417 (3)	1.02204 (9)	0.0565 (5)	
H8	0.644787	0.130582	1.027501	0.068*	
C9	0.69526 (9)	0.3306 (3)	1.04088 (10)	0.0645 (6)	
H9	0.732107	0.280446	1.058971	0.077*	
C10	0.69219 (8)	0.4939 (3)	1.03263 (8)	0.0528 (5)	
C11	0.63872 (9)	0.5713 (3)	1.00515 (9)	0.0544 (5)	
H11	0.637581	0.682263	0.999450	0.065*	
C12	0.58661 (8)	0.4804 (2)	0.98615 (8)	0.0494 (5)	
H12	0.550017	0.530722	0.967144	0.059*	
C13	0.43684 (7)	0.50197 (18)	0.83547 (6)	0.0316 (3)	
C14	0.40971 (7)	0.6175 (2)	0.85901 (7)	0.0372 (4)	
H14	0.376437	0.589579	0.868927	0.045*	
C15	0.43136 (8)	0.7731 (2)	0.86789 (8)	0.0412 (4)	
H15	0.412718	0.849067	0.883778	0.049*	
C16	0.48067 (8)	0.8167 (2)	0.85329 (8)	0.0438 (4)	
C17	0.50828 (8)	0.7033 (2)	0.83008 (9)	0.0513 (5)	
H17	0.541530	0.731489	0.820158	0.062*	
C18	0.48621 (8)	0.5466 (2)	0.82156 (8)	0.0430 (4)	
H18	0.505194	0.470334	0.806132	0.052*	
C19	0.54992 (12)	1.0247 (3)	0.85250 (17)	0.1022 (11)	
H19A	0.583775	0.962689	0.875791	0.153*	
H19B	0.557073	1.137012	0.861793	0.153*	
H19C	0.544479	1.008448	0.812661	0.153*	
C20	0.31789 (8)	0.3956 (2)	0.67329 (7)	0.0412 (4)	
C21	0.33733 (10)	0.4076 (3)	0.62226 (9)	0.0641 (6)	
H21A	0.341021	0.301250	0.608472	0.096*	
H21B	0.375514	0.461491	0.632865	0.096*	
H21C	0.307975	0.467627	0.592548	0.096*	
C22	0.31168 (8)	−0.0347 (2)	0.71859 (8)	0.0416 (4)	
C23	0.34279 (10)	−0.1848 (2)	0.71099 (9)	0.0565 (5)	
H23A	0.329284	−0.273887	0.728173	0.085*	
H23B	0.385482	−0.171936	0.729165	0.085*	
H23C	0.333649	−0.205264	0.670751	0.085*	
N1	0.39393 (6)	0.25809 (16)	0.86773 (6)	0.0352 (3)	
N2	0.74834 (9)	0.5888 (3)	1.05360 (8)	0.0727 (6)	
O1	0.41991 (6)	0.12222 (15)	0.95189 (5)	0.0462 (3)	
O2	0.48803 (5)	0.27396 (14)	0.92893 (5)	0.0394 (3)	
O3	0.79605 (8)	0.5171 (3)	1.06567 (10)	0.1097 (8)	
O4	0.74408 (9)	0.7331 (3)	1.05857 (8)	0.0893 (6)	
O5	0.49776 (7)	0.97485 (16)	0.86316 (8)	0.0654 (4)	
O6	0.36272 (5)	0.33834 (15)	0.71959 (5)	0.0410 (3)	
O7	0.26983 (6)	0.4321 (2)	0.67504 (6)	0.0632 (4)	
O8	0.34399 (5)	0.04280 (14)	0.76655 (5)	0.0441 (3)	
O9	0.26372 (7)	0.0104 (2)	0.68714 (6)	0.0684 (5)	

### Atomic displacement parameters (Å^2^)

**Table:**

	*U*^11^	*U*^22^	*U*^33^	*U*^12^	*U*^13^	*U*^23^
C1	0.0301 (8)	0.0308 (8)	0.0299 (8)	0.0021 (6)	0.0061 (6)	0.0014 (6)
C2	0.0311 (8)	0.0356 (8)	0.0303 (8)	0.0036 (6)	0.0057 (6)	−0.0026 (6)
C3	0.0270 (8)	0.0371 (9)	0.0431 (9)	0.0004 (6)	0.0039 (7)	−0.0050 (7)
C4	0.0319 (8)	0.0426 (9)	0.0435 (9)	−0.0059 (7)	0.0091 (7)	0.0002 (7)
C5	0.0390 (9)	0.0266 (7)	0.0342 (8)	0.0002 (6)	0.0075 (7)	0.0010 (7)
C6	0.0460 (10)	0.0521 (11)	0.0440 (10)	−0.0034 (9)	−0.0057 (8)	0.0169 (9)
C7	0.0402 (9)	0.0491 (10)	0.0295 (8)	−0.0012 (8)	0.0037 (7)	0.0018 (7)
C8	0.0469 (11)	0.0589 (12)	0.0545 (12)	0.0051 (9)	0.0055 (9)	0.0106 (10)
C9	0.0381 (10)	0.0846 (17)	0.0617 (13)	0.0065 (10)	0.0053 (9)	0.0104 (12)
C10	0.0394 (10)	0.0797 (15)	0.0384 (10)	−0.0140 (10)	0.0121 (8)	−0.0139 (10)
C11	0.0595 (12)	0.0502 (11)	0.0546 (12)	−0.0096 (9)	0.0207 (10)	−0.0123 (9)
C12	0.0407 (10)	0.0497 (11)	0.0518 (11)	0.0012 (8)	0.0082 (8)	−0.0019 (9)
C13	0.0315 (8)	0.0317 (8)	0.0264 (7)	−0.0002 (6)	0.0032 (6)	0.0023 (6)
C14	0.0380 (9)	0.0376 (9)	0.0369 (9)	−0.0017 (7)	0.0140 (7)	0.0002 (7)
C15	0.0452 (10)	0.0365 (9)	0.0411 (9)	0.0006 (7)	0.0139 (8)	−0.0052 (7)
C16	0.0407 (9)	0.0327 (9)	0.0521 (11)	−0.0051 (7)	0.0085 (8)	−0.0015 (8)
C17	0.0409 (10)	0.0440 (10)	0.0746 (14)	−0.0085 (8)	0.0269 (10)	−0.0028 (9)
C18	0.0395 (9)	0.0381 (9)	0.0541 (11)	0.0003 (7)	0.0195 (8)	−0.0042 (8)
C19	0.0737 (17)	0.0508 (14)	0.194 (4)	−0.0262 (13)	0.061 (2)	−0.0194 (18)
C20	0.0432 (10)	0.0369 (9)	0.0345 (9)	0.0017 (7)	0.0017 (7)	−0.0005 (7)
C21	0.0683 (14)	0.0829 (16)	0.0367 (10)	−0.0014 (12)	0.0123 (10)	0.0033 (10)
C22	0.0423 (10)	0.0399 (9)	0.0401 (9)	−0.0079 (7)	0.0110 (8)	−0.0029 (7)
C23	0.0630 (13)	0.0459 (11)	0.0595 (13)	−0.0002 (9)	0.0196 (10)	−0.0112 (9)
N1	0.0330 (7)	0.0341 (7)	0.0328 (7)	−0.0050 (6)	0.0042 (6)	0.0035 (6)
N2	0.0582 (12)	0.1074 (18)	0.0531 (11)	−0.0282 (12)	0.0200 (9)	−0.0212 (11)
O1	0.0504 (7)	0.0427 (7)	0.0422 (7)	−0.0020 (6)	0.0116 (6)	0.0132 (6)
O2	0.0364 (6)	0.0372 (6)	0.0338 (6)	−0.0042 (5)	−0.0019 (5)	0.0075 (5)
O3	0.0421 (10)	0.154 (2)	0.1255 (18)	−0.0190 (12)	0.0188 (10)	−0.0200 (15)
O4	0.0919 (13)	0.1010 (15)	0.0811 (13)	−0.0485 (12)	0.0372 (11)	−0.0322 (11)
O5	0.0564 (8)	0.0360 (7)	0.1062 (13)	−0.0139 (6)	0.0308 (8)	−0.0144 (7)
O6	0.0363 (6)	0.0518 (7)	0.0304 (6)	0.0068 (5)	0.0058 (5)	−0.0001 (5)
O7	0.0482 (8)	0.0815 (11)	0.0506 (8)	0.0230 (7)	0.0050 (6)	0.0112 (7)
O8	0.0352 (6)	0.0376 (6)	0.0502 (7)	0.0009 (5)	0.0024 (5)	−0.0093 (5)
O9	0.0561 (9)	0.0722 (10)	0.0559 (9)	0.0083 (8)	−0.0077 (7)	−0.0200 (8)

### Geometric parameters (Å, º)

**Table:**

C1—N1	1.468 (2)	C12—H12	0.9300
C1—C13	1.507 (2)	C13—C18	1.376 (2)
C1—C2	1.536 (2)	C13—C14	1.388 (2)
C1—H1	0.9800	C14—C15	1.377 (2)
C2—O6	1.4293 (19)	C14—H14	0.9300
C2—C3	1.516 (2)	C15—C16	1.381 (3)
C2—H2	0.9800	C15—H15	0.9300
C3—O8	1.444 (2)	C16—O5	1.369 (2)
C3—C4	1.511 (2)	C16—C17	1.378 (3)
C3—H3	0.9800	C17—C18	1.388 (3)
C4—N1	1.472 (2)	C17—H17	0.9300
C4—H4A	0.9700	C18—H18	0.9300
C4—H4B	0.9700	C19—O5	1.409 (3)
C5—O1	1.214 (2)	C19—H19A	0.9600
C5—N1	1.344 (2)	C19—H19B	0.9600
C5—O2	1.3476 (19)	C19—H19C	0.9600
C6—O2	1.4371 (19)	C20—O7	1.191 (2)
C6—C7	1.496 (3)	C20—O6	1.354 (2)
C6—H6A	0.9700	C20—C21	1.488 (3)
C6—H6B	0.9700	C21—H21A	0.9600
C7—C8	1.382 (3)	C21—H21B	0.9600
C7—C12	1.385 (3)	C21—H21C	0.9600
C8—C9	1.373 (3)	C22—O9	1.195 (2)
C8—H8	0.9300	C22—O8	1.337 (2)
C9—C10	1.368 (3)	C22—C23	1.490 (3)
C9—H9	0.9300	C23—H23A	0.9600
C10—C11	1.372 (3)	C23—H23B	0.9600
C10—N2	1.476 (3)	C23—H23C	0.9600
C11—C12	1.382 (3)	N2—O3	1.218 (3)
C11—H11	0.9300	N2—O4	1.210 (3)
			
N1—C1—C13	115.20 (13)	C18—C13—C1	120.44 (15)
N1—C1—C2	101.03 (12)	C14—C13—C1	121.24 (14)
C13—C1—C2	111.89 (12)	C15—C14—C13	120.91 (16)
N1—C1—H1	109.5	C15—C14—H14	119.5
C13—C1—H1	109.5	C13—C14—H14	119.5
C2—C1—H1	109.5	C14—C15—C16	120.28 (16)
O6—C2—C3	115.70 (13)	C14—C15—H15	119.9
O6—C2—C1	108.19 (12)	C16—C15—H15	119.9
C3—C2—C1	105.25 (13)	O5—C16—C17	125.10 (17)
O6—C2—H2	109.2	O5—C16—C15	115.33 (17)
C3—C2—H2	109.2	C17—C16—C15	119.56 (16)
C1—C2—H2	109.2	C16—C17—C18	119.65 (17)
O8—C3—C4	107.94 (14)	C16—C17—H17	120.2
O8—C3—C2	109.73 (13)	C18—C17—H17	120.2
C4—C3—C2	101.92 (13)	C13—C18—C17	121.37 (17)
O8—C3—H3	112.2	C13—C18—H18	119.3
C4—C3—H3	112.2	C17—C18—H18	119.3
C2—C3—H3	112.2	O5—C19—H19A	109.5
N1—C4—C3	103.88 (13)	O5—C19—H19B	109.5
N1—C4—H4A	111.0	H19A—C19—H19B	109.5
C3—C4—H4A	111.0	O5—C19—H19C	109.5
N1—C4—H4B	111.0	H19A—C19—H19C	109.5
C3—C4—H4B	111.0	H19B—C19—H19C	109.5
H4A—C4—H4B	109.0	O7—C20—O6	122.57 (17)
O1—C5—N1	124.23 (15)	O7—C20—C21	126.14 (17)
O1—C5—O2	124.12 (15)	O6—C20—C21	111.29 (16)
N1—C5—O2	111.62 (14)	C20—C21—H21A	109.5
O2—C6—C7	109.81 (15)	C20—C21—H21B	109.5
O2—C6—H6A	109.7	H21A—C21—H21B	109.5
C7—C6—H6A	109.7	C20—C21—H21C	109.5
O2—C6—H6B	109.7	H21A—C21—H21C	109.5
C7—C6—H6B	109.7	H21B—C21—H21C	109.5
H6A—C6—H6B	108.2	O9—C22—O8	123.71 (17)
C8—C7—C12	119.00 (18)	O9—C22—C23	125.35 (17)
C8—C7—C6	118.12 (17)	O8—C22—C23	110.92 (15)
C12—C7—C6	122.73 (17)	C22—C23—H23A	109.5
C9—C8—C7	120.8 (2)	C22—C23—H23B	109.5
C9—C8—H8	119.6	H23A—C23—H23B	109.5
C7—C8—H8	119.6	C22—C23—H23C	109.5
C10—C9—C8	119.0 (2)	H23A—C23—H23C	109.5
C10—C9—H9	120.5	H23B—C23—H23C	109.5
C8—C9—H9	120.5	C5—N1—C1	124.66 (13)
C9—C10—C11	121.97 (19)	C5—N1—C4	119.39 (14)
C9—C10—N2	118.7 (2)	C1—N1—C4	112.69 (12)
C11—C10—N2	119.3 (2)	O3—N2—O4	124.1 (2)
C10—C11—C12	118.5 (2)	O3—N2—C10	118.1 (2)
C10—C11—H11	120.8	O4—N2—C10	117.8 (2)
C12—C11—H11	120.8	C5—O2—C6	113.56 (13)
C11—C12—C7	120.73 (18)	C16—O5—C19	118.13 (17)
C11—C12—H12	119.6	C20—O6—C2	116.07 (13)
C7—C12—H12	119.6	C22—O8—C3	117.36 (13)
C18—C13—C14	118.23 (15)		
			
N1—C1—C2—O6	−155.47 (12)	C15—C16—C17—C18	0.1 (3)
C13—C1—C2—O6	81.46 (16)	C14—C13—C18—C17	−0.7 (3)
N1—C1—C2—C3	−31.22 (15)	C1—C13—C18—C17	175.80 (16)
C13—C1—C2—C3	−154.29 (13)	C16—C17—C18—C13	0.5 (3)
O6—C2—C3—O8	44.85 (17)	O1—C5—N1—C1	−166.43 (16)
C1—C2—C3—O8	−74.51 (15)	O2—C5—N1—C1	15.2 (2)
O6—C2—C3—C4	159.06 (13)	O1—C5—N1—C4	−8.4 (3)
C1—C2—C3—C4	39.70 (16)	O2—C5—N1—C4	173.30 (14)
O8—C3—C4—N1	83.80 (15)	C13—C1—N1—C5	−68.82 (19)
C2—C3—C4—N1	−31.73 (16)	C2—C1—N1—C5	170.43 (14)
O2—C6—C7—C8	−148.04 (18)	C13—C1—N1—C4	131.85 (14)
O2—C6—C7—C12	36.3 (3)	C2—C1—N1—C4	11.10 (16)
C12—C7—C8—C9	1.3 (3)	C3—C4—N1—C5	−147.44 (15)
C6—C7—C8—C9	−174.4 (2)	C3—C4—N1—C1	13.09 (18)
C7—C8—C9—C10	0.1 (3)	C9—C10—N2—O3	16.0 (3)
C8—C9—C10—C11	−1.2 (3)	C11—C10—N2—O3	−163.5 (2)
C8—C9—C10—N2	179.30 (19)	C9—C10—N2—O4	−162.9 (2)
C9—C10—C11—C12	0.9 (3)	C11—C10—N2—O4	17.7 (3)
N2—C10—C11—C12	−179.61 (18)	O1—C5—O2—C6	7.0 (2)
C10—C11—C12—C7	0.6 (3)	N1—C5—O2—C6	−174.71 (15)
C8—C7—C12—C11	−1.7 (3)	C7—C6—O2—C5	−168.20 (15)
C6—C7—C12—C11	173.92 (19)	C17—C16—O5—C19	−4.5 (3)
N1—C1—C13—C18	137.44 (16)	C15—C16—O5—C19	176.2 (2)
C2—C1—C13—C18	−107.93 (17)	O7—C20—O6—C2	−4.1 (2)
N1—C1—C13—C14	−46.17 (19)	C21—C20—O6—C2	175.16 (15)
C2—C1—C13—C14	68.47 (19)	C3—C2—O6—C20	85.09 (17)
C18—C13—C14—C15	0.4 (2)	C1—C2—O6—C20	−157.16 (13)
C1—C13—C14—C15	−176.07 (15)	O9—C22—O8—C3	2.4 (3)
C13—C14—C15—C16	0.1 (3)	C23—C22—O8—C3	−176.13 (15)
C14—C15—C16—O5	179.04 (16)	C4—C3—O8—C22	141.18 (15)
C14—C15—C16—C17	−0.4 (3)	C2—C3—O8—C22	−108.54 (16)
O5—C16—C17—C18	−179.27 (19)		

### Hydrogen-bond geometry (Å, º)

**Table:**

*D*—H···*A*	*D*—H	H···*A*	*D*···*A*	*D*—H···*A*
C4—H4*B*···O7^i^	0.97	2.60	3.129 (2)	115
C6—H6*A*···O1^ii^	0.97	2.54	3.250 (2)	130

Symmetry codes: (i) −*x*+1/2, *y*−1/2, −*z*+3/2; (ii) −*x*+1, −*y*, −*z*+2.

## References

[bb1] Brandenburg, K. (2006). *DIAMOND*. Crystal Impact GbR, Bonn, Germany.

[bb2] Burla, M. C., Caliandro, R., Carrozzini, B., Cascarano, G. L., Cuocci, C., Giacovazzo, C., Mallamo, M., Mazzone, A. & Polidori, G. (2015). *J. Appl. Cryst.* **48**, 306–309.

[bb3] ChemAxon (2010). *Marvinsketch.* http://www.chemaxon.com.

[bb4] Coleman, R. S., Felpin, F.-X. & Chen, W. (2004). *J. Org. Chem.* **69**, 7309–7316. 10.1021/jo048924i 15471485

[bb5] Contreras-García, J., Johnson, E. R., Keinan, S., Chaudret, R., Piquemal, J.-P., Beratan, D. N. & Yang, W. (2011). *J. Chem. Theory Comput.* **7**, 625–632. 10.1021/ct100641a PMC3080048 21516178

[bb6] Dallasta Pedroso, S., Caracelli, I., Zukerman-Schpector, J., Soto-Monsalve, M., De Almeida Santos, R. H., Correia, C. R. D., Llanes Garcia, A. L., Kwong, H. C. & Tiekink, E. R. T. (2020). *Acta Cryst.* E**76**, 967–972. 10.1107/S205698902000701X PMC7273984 32523774

[bb7] Dhameja, M. & Gupta, P. (2019). *Eur. J. Med. Chem.* **176**, article No. 343e377. 10.1016/j.ejmech.2019.04.025 31112894

[bb8] Enraf–Nonius (1989). *CAD-4 EXPRESS*. Enraf–Nonius, Delft, The Netherlands.

[bb9] Farrugia, L. J. (2012). *J. Appl. Cryst.* **45**, 849–854.

[bb10] Garcia, A. L. L. (2008). PhD thesis, Universidade Estadual de Campinas, UNICAMP, Campinas, SP, Brazil.

[bb11] Harms, K. & Wocadlo, S. (1995). *XCAD4*. University of Marburg, Germany.

[bb12] Johnson, E. R., Keinan, S., Mori-Sánchez, P., Contreras-García, J., Cohen, A. J. & Yang, W. (2010). *J. Am. Chem. Soc.* **132**, 6498–6506. 10.1021/ja100936w PMC2864795 20394428

[bb13] Kiappes, J. L., Hill, M. L., Alonzi, D. S., Miller, J. L., Iwaki, R., Sayce, A. C., Caputo, A. T., Kato, A. & Zitzmann, N. (2018). *Chem. Biol.* **13**, 60–65. 10.1021/acschembio.7b00870 PMC5824344 29161006

[bb14] Qian, B.-C., Kamori, A., Kinami, K., Kato, A., Li, Y.-X., Fleet, G. W. J. & Yu, C.-Y. (2016). *Org. Biomol. Chem.* **14**, 4488–4498. 10.1039/c6ob00531d 27093691

[bb15] Sheldrick, G. M. (1996). *SADABS*. University of Göttingen, Germany.

[bb16] Sheldrick, G. M. (2015). *Acta Cryst.* C**71**, 3–8.

[bb17] Spek, A. L. (2020). *Acta Cryst.* E**76**, 1–11. 10.1107/S2056989019016244 PMC6944088 31921444

[bb18] Tan, S. L., Jotani, M. M. & Tiekink, E. R. T. (2019). *Acta Cryst.* E**75**, 308–318. 10.1107/S2056989019001129 PMC6399703 30867939

[bb19] Taylor, C. M. & Weir, C. A. (2000). *J. Org. Chem.* **65**, 1414–1421. 10.1021/jo991523w 10814103

[bb20] Turner, M. J., Mckinnon, J. J., Wolff, S. K., Grimwood, D. J., Spackman, P. R., Jayatilaka, D. & Spackman, M. A. (2017). *Crystal Explorer 17*. The University of Western Australia.

[bb21] Westrip, S. P. (2010). *J. Appl. Cryst.* **43**, 920–925.

[bb22] Zukerman-Schpector, J., Sugiyama, F. H., Garcia, A. L. L., Correia, C. R. D., Jotani, M. M. & Tiekink, E. R. T. (2017). *Acta Cryst.* E**73**, 1218–1222. 10.1107/S2056989017009987 PMC5598852 28932440

